# Editorial: World refugee day 2023

**DOI:** 10.3389/fpubh.2024.1513414

**Published:** 2024-12-11

**Authors:** Ahmed Hossain, Shela Akbar Ali Hirani, Silvia Candela, Mimoza Lika Shahini, Stefano Orlando

**Affiliations:** ^1^College of Health Sciences, University of Sharjah, Sharjah, United Arab Emirates; ^2^Faculty of Nursing, University of Regina, Regina, SK, Canada; ^3^Local Health Authority of Reggio Emilia-IRCCS, Reggio Emilia, Italy; ^4^University Clinical Center of Kosovo, Prishtine, Albania; ^5^Department of Biomedicine and Prevention, University of Rome Tor Vergata, Rome, Italy

**Keywords:** refugee population, displaced individuals, migration, health, mental health, healthcare services

Forced displacement continues to be a major global issue, affecting 117.3 million people by the end of 2023. Of these, 31.6 million are refugees, driven from their homes by conflict, natural disasters, persecution, and environmental crises (1). Refugees are disproportionately affected by conflicts and crises, facing a decline in quality of life, heightened exposure to violence, and increased health risks (2, 3). They often lack access to essential needs such as healthcare, food, water, and support, which significantly impact their wellbeing. This highlights the need for shared responsibility to ensure their protection and dignity (4). Rebuilding healthcare systems after conflicts and disasters can take years, leading to poor health outcomes, food insecurity, and intergenerational trauma.

Earlier in 2023, a Research Topic of articles was published on the health of displaced people (5). A dedicated edition of Frontiers in Public Health focusing on the factors that improve the health of refugee populations is essential. The Research Topic “*World Refugee Day 2023*” centers on factors that improve refugee health. The published articles identify indicators of health, healthcare, mental health, and wellbeing to enhance global refugee healthcare outcomes. Key contributions are summarized below.

Phung explored the challenges and recommendations for caring for resettled refugee children in the United States, emphasizing the need for culturally sensitive, trauma-informed care. The discussion underscores the importance of addressing language barriers and mental health concerns. Additionally, the paper highlighted the role of public health in preventing infectious diseases, promoting mental wellbeing, and delivering health education.

Choudhary et al. examined the prevalence of stunting among refugee and internally displaced children under the age of 5 years, revealing significantly higher rates than the global average. The study also highlighted geographical disparities, with stunting rates notably higher in regions such as Africa and Southeast Asia.

Six articles were published in the European context, specifically addressing the need to improve health and mental health literacy, and healthcare services, for refugee populations. Kordel et al. investigated the prevalence of acute stress disorder (ASD) among Ukrainian refugees displaced by the 2022 war, revealing a high rate of ASD and underscoring the severe psychological toll of the conflict. The study identified key risk factors for ASD, including witnessing violence, separation from loved ones, and preexisting mental health conditions. Gerber et al. examined the relationship between overweight, cardiovascular risk markers, and mental health in forcibly displaced individuals in a Greek refugee camp, finding that higher cardiorespiratory fitness levels help to mitigate the negative effects of overweight and cardiovascular risks on mental health.

Portela et al. analyzed refugees' access to healthcare services in Lisbon, Portugal, during the COVID-19 pandemic, highlighting barriers such as language difficulties, limited healthcare system knowledge, and financial constraints. The study emphasized the importance of culturally sensitive, linguistically appropriate healthcare services for refugee populations. Ekblad et al. conducted a group intervention with separated Ukrainian refugee families in Sweden aimed at improving perceived health and mental health literacy. The intervention successfully enhanced participants' understanding of health issues and boosted their confidence in accessing healthcare services.

Cimino et al. assessed the knowledge and skills of general practitioner trainees in Sicily regarding global competency standards for health workers dealing with refugee and migrant health. The findings highlighted the need for improved training and education to equip healthcare professionals with the necessary skills to support vulnerable populations. El Arab et al. explored the health and social needs of asylum seekers and undocumented migrants crossing from Belarus to Lithuania. Through qualitative interviews, the study revealed urgent needs for healthcare, shelter, and social support services, with participants expressing frustration over bureaucratic processes and restricted mobility. The research highlighted the critical need for comprehensive, culturally appropriate support for these vulnerable groups.

Four articles focused on the mental health of refugee populations. Ermansons et al. explored the mental health of Somali refugees in urban neighborhoods using an eco-social approach to examine how trauma, social isolation, and economic hardship affect their wellbeing. The study emphasized the importance of social support networks and community-based interventions. Cherepanov stressed the need for a politically informed approach to refugee healthcare, arguing that political experiences, such as trauma and discrimination, profoundly impact mental health. The study advocated for incorporating political competencies alongside cultural sensitivity and trauma-informed care to address ethical challenges in refugee healthcare. Manafe et al. examined the prevalence and factors contributing to common mental disorders, such as depression and anxiety, among internally displaced people (IDPs) in Cabo Delgado, Mozambique. The study found high rates of these disorders linked to violence, loss, and displacement stressors. Assaf et al. presented a framework to understand the mental health challenges of Syrian refugees, focusing on prewar, displacement, and post-displacement stressors. The framework emphasized the cumulative impact of these experiences and the need for tailored mental health interventions.

Hossain stressed the urgent need to provide healthcare services to displaced individuals worldwide, as more people are forced to flee due to conflict, persecution, and natural disasters. Access to healthcare is crucial for preventing disease, treating injuries, and ensuring wellbeing in displaced populations. Liu et al. explored the impact of social integration on older migrants' access to health services in China, using national data. The study found that greater social integration is linked to the increased use of community-based healthcare facilities, highlighting the importance of integration in improving healthcare access for older migrants.

Kvasnevska et al. explored the link between war-related factors and the spread of sexually transmitted infections (STIs), conducting a systematic review of the literature to identify key contributors to rising STI rates in conflict zones. The study highlighted how displacement, sexual violence, economic hardship, and healthcare disruptions increase STI transmission among vulnerable groups. It underscored the importance of targeted interventions to address these factors and curb the spread of STIs in war-affected areas.

Across the various studies, common key points emerged regarding the healthcare challenges faced by refugees and displaced populations. These included the critical need for culturally sensitive and trauma-informed healthcare, the importance of addressing mental health through both community-based and policy approaches, and the significant barriers that refugees face in accessing healthcare services, such as language difficulties, financial constraints, and lack of knowledge about healthcare systems. Social integration and fitness were also highlighted as important factors that can enhance health outcomes for displaced individuals.

Several key recommendations were proposed to enhance refugee health and foster resilient communities. These include implementing mandatory cultural competence and trauma-informed care training for healthcare providers and ensuring access to qualified interpreters and translation services for effective communication. Developing multilingual, culturally tailored health education materials is essential. Additional recommendations include integrating mental health screenings into routine care, expanding culturally competent mental health services, and streamlining enrollment processes for timely access. Mental health services must be prioritized, with an emphasis on community support and integration programs to mitigate the effects of trauma and social isolation. Establishing navigation programs will assist refugees in navigating complex healthcare systems. Partnerships with community organizations, robust immunization programs, and nutrition education will further support refugee needs. Advocacy for policies that ensure equitable access to healthcare, housing, employment, and education is vital for strengthening refugee health and community resilience. Additionally, targeted interventions are essential for addressing specific health issues, such as sexually transmitted infections and chronic diseases that are exacerbated by displacement.

While some studies have addressed mental health, further investigation into how it transmits across generations and affects refugee children is needed. The impact of chronic stress on physical health, such as cardiovascular issues and autoimmune disorders among refugees, also requires further study. Developing culturally appropriate mental health interventions, especially for women's health, gender-based violence, and maternal care, is crucial. Research should examine the health challenges faced by older refugees, including chronic diseases and social isolation, as well as healthcare access for disabled refugees. Telehealth and mobile health apps could improve healthcare for refugees, especially in remote areas. Additionally, understanding climate change impacts, policy gaps, and healthcare system limitations will aid in addressing refugees' complex health needs.
